# Maternal Blood Manganese and Early Neurodevelopment: The Mothers and Children’s Environmental Health (MOCEH) Study

**DOI:** 10.1289/ehp.1307865

**Published:** 2015-03-03

**Authors:** Soo Eun Chung, Hae-Kwan Cheong, Eun-Hee Ha, Boong-Nyun Kim, Mina Ha, Yangho Kim, Yun-Chul Hong, Hyesook Park, Se-Young Oh

**Affiliations:** 1 Department of Social and Preventive Medicine, Sungkyunkwan University School of Medicine, Suwon, Republic of Korea; 2 Center for Molecular Medicine, Samsung Biomedical Research Institute, Seoul, Republic of Korea; 3 Department of Preventive Medicine, Ewha Womans University School of Medicine, Seoul, Republic of Korea; 4 Department of Neuropsychiatry, Seoul National University Hospital, Seoul, Republic of Korea; 5 Department of Psychiatry, Seoul National University College of Medicine, Seoul, Republic of Korea; 6 Department of Preventive Medicine, Dankook University College of Medicine, Cheonan, Republic of Korea; 7 Department of Occupational and Environmental Medicine, College of Medicine, Ulsan University, Ulsan, Republic of Korea; 8 Department of Preventive Medicine, Seoul National University College of Medicine, Seoul, Republic of Korea; 9 Department of Food and Nutrition, Kyung Hee University College of Human Ecology, Seoul, Republic of Korea

## Abstract

**Background:**

Manganese is an essential trace element and common component of water, soil, and air. Prenatal manganese exposure may affect fetal and infantile neurodevelopment, but reports on *in utero* manganese exposure and infant neurodevelopment are rare.

**Objective:**

This study was conducted to investigate a relationship between maternal blood manganese level and neurodevelopment of infants at 6 months of age.

**Methods:**

Data were obtained from the Mothers and Children’s Environmental Health (MOCEH) birth cohort study. The study population included 232 pairs of pregnant women and their infants at 6 months of age. Maternal blood manganese was measured at term, just before delivery. Mental and psychomotor development in infancy was assessed at 6 months of age using the Bayley Scales of Infant Development. The relationship between maternal blood manganese level and the mental and psychomotor development indexes (MDI and PDI) was estimated for manganese modeled as a linear and as a categorical variable and using penalized splines for nonlinear modeling.

**Results:**

Mean ± SD maternal blood manganese concentration was 22.5 ± 6.5 μg/L. After adjustment for potential confounders, blood manganese was used as a continuous variable in a linear and nonlinear model. Associations between maternal blood manganese and MDI and PDI scores followed an inverted U-shape dose–response curve after adjustment for potential confounders, with lower scores associated with both low and high blood concentrations [MDI: likelihood-ratio test (LRT) *p* = 0.075, PDI: LRT *p* = 0.038]. Associations of both outcomes with increasing blood manganese shifted from positive to negative at concentrations of 24–28 μg/L in this cohort of term, normal birth weight children.

**Conclusion:**

Although no cut-off point has been established to define manganese toxicity, both high and low blood manganese levels may be associated with neurobehavioral function in infants.

**Citation:**

Chung SE, Cheong HK, Ha EH, Kim BN, Ha M, Kim Y, Hong YC, Park H, Oh SY. 2015. Maternal blood manganese and early neurodevelopment: the Mothers and Children’s Environmental Health (MOCEH) study. Environ Health Perspect 123:717–722; http://dx.doi.org/10.1289/ehp.1307865

## Introduction

Manganese is recognized as a human neurotoxicant when originating from occupational or environmental exposure, despite being an essential element. Manganese neurotoxicity has been associated with dopaminergic dysregulation and inhibition of its metabolism (Guilarte 2013). The severe form of human neurotoxicity to manganese resulting from occupational exposure typically manifests as chronic manganism (Kim et al. 1998; Lucchini et al. 1999), a variant form of parkinsonism. In children, manganese exposure has been associated with the prevalence of attention deficit hyperactivity disorder (ADHD) (Bouchard et al. 2007, 2011; Woolf et al. 2002) and negatively associated with IQ scores (Wasserman et al. 2006; Wright et al. 2006). There have been recent reports of negative associations between exposure to manganese and cognition, memory, and motor function in children (Bouchard et al. 2011; Khan et al. 2012; Lucchini et al. 2012; Menezes-Filho et al. 2011; Meyer-Baron et al. 2013; Takser et al. 2003; Wasserman et al. 2006).

We hypothesize that the role of manganese during early stages of development may be related primarily to its biological activity as an essential nutrient in human metabolism. Manganese is crucial for a number of biological and physiological processes, including body growth, immune function, enzymatic regulation reactions, bone growth and metabolism (Agency for Toxic Substances and Disease Registry 2012; Yoon et al. 2011). Manganese can cross a placenta during pregnancy to reach a developing fetus (Erikson et al. 2007; Spencer 1999). During pregnancy, the maternal blood manganese level rises, especially after the second trimester, with the highest level manifested in cord blood (Yoon et al. 2011). Relatively higher maternal blood level of manganese suggests increased biological demand during pregnancy and its biological role in fetal development (Tholin et al. 1995).

There have been few published studies of associations between neurodevelopmental outcomes and maternal blood manganese levels during pregnancy or children’s blood levels in early life. In a cohort study, blood manganese concentrations at 12 months of age showed an inverted U-shaped association with developmental function at 12 and 24 months (Claus Henn et al. 2010). Cord blood manganese above the 75th percentile was negatively associated with neurodevelopment scores at age 2 years (Lin et al. 2013). A recent nationwide cross-sectional study of 1,089 8- to 11-year-old children in Korea also reported that both high and low blood manganese levels were associated with lower scores for attention and cognition (Bhang et al. 2013). Our results support the need for additional studies of the nonlinear relationship between blood manganese levels and neurodevelopment.

In this study, we aimed to evaluate the relationship between neurodevelopment and maternal blood manganese level in a birth cohort without a specific source of occupational or environmental exposure. We investigated the relationship between both lower and higher level of maternal blood manganese and neurodevelopment.

## Subjects and Methods

*Study subjects*. Participants were recruited from the Mothers and Children’s Environmental Health (MOCEH) study, a birth cohort study designed to assess the association of pre- and postnatal environmental exposures on growth, development, and health outcomes from early fetal life to young adulthood. Participants were recruited from three university hospitals located in Seoul (metropolitan area), Ulsan (metropolitan and industrial area, southeast), and Cheonan (urban area, midwest), Republic of Korea (Kim et al. 2009).

MOCEH participants were recruited from 2007 to 2011 and included pregnant women in the first trimester who were newly registered at a local center during their visit. Mothers, and spouses whenever possible, were informed of the study and voluntarily decided to participate. Blood samples for manganese measurements were obtained from mothers recruited between July 2007 and January 2009. Among the total of 667 mothers recruited by the end of 2011, maternal blood samples were obtained from 352 mothers. A total of 265 children had maternal blood samples collected during pregnancy and neurodevelopmental testing at 6 months of age. There were no significant differences between mothers with (*n* = 265) and without (*n* = 87) measurement of infants’ Bayley Scales of Infant Development-II (BSID-II) scores at 6 months with respect to maternal blood manganese concentration, mother’s age, residential area, education level, and the birth weight of the children (data not shown). Children were excluded if they were born from pregnancies that lasted < 37 weeks or > 42 weeks, which includes 3 infants with birth weight < 2,500 g (*n* = 27), twin (*n* = 2), or if data on main confounding variables were missing or incomplete (*n* = 4). The final number of study subjects was 232 mother–infant pairs.

The study protocol, including all materials and procedures, was approved by the institutional review boards of Ewha Womans University Hospital, Dankook University Hospital, and Ulsan University Hospital. Eligible pregnant women were informed about the study, and written consent was obtained before participation.

*Measurements.* Questionnaire surveys were conducted more than three times: at the time of recruitment, at the visit for delivery, and at each time of the infant’s follow-up visit. Participants were interviewed by a trained interviewer during their visit to a local center. The questionnaire included general information on demographic and socioeconomic factors and other potential confounders including maternal age, height, weight, maternal and paternal education level, marital status at the time of enrollment, and family income. The subjects were asked to describe their entire food intake during prior 24 hr before the interview and the total calorie intake was calculated using nutrient intake assessment software (CAN-Pro 3.0; Korean Nutrition Society, Seoul, Korea). Data collected before delivery included exposure to passive smoking at home, the parents’ physical condition, their medical records, and family history of diseases.

Information on birth outcome including date of delivery, mode of delivery, birth weight and height, gestational age, head circumference at birth, parity, and infant’s sex were collected from the medical records at delivery. Information regarding variables that could affect the infants’ growth after birth (colostrum feeding, household income, and nutritional supplements) was collected by a survey during the postnatal hospital visit (Claus Henn et al. 2010; Kim et al. 2009; Takser et al. 2004; Zota et al. 2009). Trained nurses in the delivery room routinely measured birth weights using a digital scale at birth and recorded it on the mothers’ medical records.

Follow-up visits were planned every 6 months after birth until 3 years of age. The infant’s weight, height, and head circumference were measured at 6 months to evaluate growth in addition to the assessment of neurodevelopment.

*Blood manganese assessment*. A venous whole blood sample (15 mL) was collected in a trace metal–free tube from pregnant women before delivery when they visited for delivery. Blood samples were immediately frozen and stored at –70°C until analysis. Manganese concentration was measured with a graphite furnace–atomic absorption spectrometer (AAnalyst600; Perkin Elmer, Waltham, MA, USA) according to the quality control measures of the Korean Society for Laboratory Medicine (KSLM) and College of American Pathologists (KSLM 2010). Quality control for manganese was performed using a Levey–Jennings chart, with the mean ± SD values set as the allowable range. All maternal blood manganese analyses were carried out by Special Chemistry Center, Seegene Medical Foundation (former Neodin Medical Institute, Seoul, Korea). For the internal quality assurance and control program, commercial reference materials were obtained from Seronorm human whole blood (Sero Ltd, Billingstad, Norway). As part of the external quality assurance and control, the institute passed the German External Quality Assessment Scheme (G-EQUAS; http://www.g-equas.de) operated by Friedrich-Alexander University and also passed the Quality Assurance Program operated by the Korea Occupational Safety and Health Agency (http://msds.kosha.or.kr). Precision (percent relative standard deviation) was < 5% and all samples were above the limits of detection. The average limit of detection of maternal blood manganese was 1.585 μg/L.

*BSID-II.* Infant neurodevelopment was assessed according to the BSID-II for neurodevelopment, from 0 to 3 years of age (Bayley 1993). In the MOCEH study, Korean version of the BSID-II was applied at 6, 12, 24, and 36 months of age (Kim et al. 2009). The Korean version of the BSID-II was validated by back translation and test–retest stability (Park and Cho 2006). The BSID-II was conducted in a quiet room by trained examiners for 30–45 min, being attended by a parent. The BSID-II consists of developmental tasks that assess mental development, motor abilities, and postural control. Each test was standardized to produce developmental indexes with a mean score of 100 and an SD of 15 (a composite score that compares the child’s developmental performance with the norms for typically developing Korean children of the same age). The specific indexes were the mental development index (MDI) and the psychomotor development index (PDI) (Bayley 1993). All test (include video monitoring of the examination) procedures and inter-rater reliability (kappa value > 0.8) were conducted according to the Standards for Educational and Psychological Testing (American Educational Research Association et al. 1999). Each measurement was double-checked and confirmed through feedback between the examiner and the central coordinator.

*Statistical analysis*. We evaluated the distribution of demographic, socioeconomic, and other factors potentially related to manganese and neurodevelopment such as maternal and infant’s medical information at delivery and at the 6-month follow-up. Neurodevelopment scores were approximately normally distributed and were modeled as continuous variables. Possible associations of selected potential confounders (maternal age, gestational period, monthly income, breastfeeding status, maternal total calorie intake, infant birth order, residential area, infant sex, and birth weight) with maternal blood manganese and neurodevelopment score (MDI and PDI) were explored separately with bivariate regression. The list of potential confounders was based on biological plausibility by literature review and was tested in the final model based on statistical significance and changes in *R*^2^ (coefficent of determination) level. Level of significance was *p* < 0.1 for each variable, but variables that are essential in the assessment of fetal and infant development, such as gestational age and birth weight, sex of the baby, and maternal age, were included in the model even if they were not significant. Variables that have a high contribution to the model, such as parity and total calorie intake, were included in the model for their contribution to the *R*^2^ in either MDI or PDI model. The final set of confounders comprised maternal age, gestation period (days), monthly income, breastfeeding status, maternal total calorie intake (kilocalories/day), infant birth order, residential area, infant sex, and birth weight (kilograms) (Table 1). Confounders that are continuous in nature, such as gestational period, maternal calorie intake, and birth weight, were included in the model in continuous variables. Missing variables were included in the model by categorizing into a separate category of missing.

**Table 1:** Characteristics of the study participants (*n* = 232) and distribution of maternal blood manganese concentration at delivery and postnatal MDI/PDI scores.

Characteristic	*n* (%) or mean ± SD
Mothers
Maternal age (years)	30.1 ± 3.5
Gestation period (days)	276 ± 7.1
Maternal educational level
High school and below	93 (40.2)
College and above	138 (59.8)
Missing	1
Monthly income (US$)^*a*^
< 2,000	58 (26.1)
2,000–2,999	83 (37.4)
≥ 3,000	81 (36.5)
Missing	10
Area
Seoul	58 (25.0)
Cheonan	37 (15.9)
Ulsan	137 (59.1)
Breastfeeding
No	97 (52.4)
Yes	88 (47.6)
Missing	47
Birth order
1st	131 (56.7)
≥ 2nd	100 (43.3)
Missing	1
Total calorie intake during pregnancy [kcal/day (range)]	1,772 ± 496 (603–4,004)
Infants
Sex	
Male	108 (46.6)
Female	124 (53.4)
Body weight [kg (range)]	
At birth	3.3 ± 0.3 (2.6–4.3)
At 6 months	8.5 ± 0.9 (6.0–12.3)
Blood manganese (μg/L)	22.5 ± 6.5
Bayley MDI score	94.4 ± 11.7
Bayley PDI score	93.4 ± 14.3
^***a***^1 US$ = 1,100 KRW (as of 2009).	

A linear regression model was applied for the concentration of mother’s manganese and 6-month MDI and PDI. In the simple linear model (model 1), a linear relationship was modeled with the infants’ developmental indexes as response variables and the maternal blood manganese as a predictor variable after adjustment for the confounders. We also ran a quadratic model that included manganese and squared manganese as predictor variables of the nonlinear model.

To analyze the dose–response relationship between the maternal blood manganese concentrations and BSID-II scores, we divided all participants into four groups at intervals of 5 μg/L (< 20 μg/L, 20–24 μg/L, 25–29 μg/L, ≥ 30 μg/L). In addition, we conducted multivariable regression between the four manganese groups as independent variables and MDI or PDI score as the dependent variables after controlling for possible confounders.

We used generalized additive models (GAM) and the generalized linear model (GLM) with a linear predictor involving a sum of smoothing functions of covariates to estimate the relationship between maternal blood manganese and neurodevelopmental indexes, while controlling for confounders. We used a series of adjusted models to evaluate the shape of the dose–response relation between manganese and the outcomes, including models of manganese as a simple linear variable, quadratic models that included terms for manganese and manganese squared, and GAMs using penalized spline-smoothing functions to estimate associations of manganese with the neurodevelopmental outcomes. We modeled manganese as a categorical variable (< 20 μg/L, 20–24 μg/L, 25–29 μg/L, ≥ 30 μg/L) and used spline terms for variable with blood manganese and covariate with birth weight in the GAM models.

We used the equation E(Y) = β_0_ + β × NS(blood Mn) + NS(birth weight) + factor(residential area) + factor(infant sex) + maternal age + gestational period + monthly income + infant birth order, where E(Y) was the expected value of neurodevelopmental indexes (MDI/PDI), β_0_ was the intercept, β was the coefficient of maternal blood manganese, NS was the smoothing functions, and the other factors were the confounders in categorical variables. To determine point of transition, we used a stepwise method to select the best fit model with the smallest Akaike’s information criterion (AIC) value (Hastie and Tibshirani 1990).

We estimated the inflection point of maternal blood manganese concentration and neurodevelopment score using piecewise regression. A piecewise regression model, which assumes that two lines are joined at unknown points, can be used to estimate inflection point to determine the width of edge effects for smoothing (Seber and Wild 2003). The inflection point was determined using trial and error, by selecting turning points along a predefined interval and then choosing the turning point that resulted in the maximum model likelihood indicated by AIC.

For all analyses, probability values < 0.5 were considered statistically significant. Statistical analyses were conducted using SAS version 9.3 (SAS Institute Inc., Cary, NC, USA) and R version 2.14.2 (R Core Team 2014). The blood manganese inflection point was determined with the HEAT package in R. Results were considered statistically significant at *p* < 0.05.

## Results

*General characteristics of the subjects*. The maternal participants had a mean ± SD age of 30.1 ± 3.5 years and mean gestational period at delivery of 276 ± 7.1 days. Maternal educational level was high: 59% of the participants graduated from college or postgraduate studies. The children had a mean birth weight of 3.3 ± 0.3 kg, and 46.6% were boys. The major demographic characteristics of the participants were summarized in Table 1.

*Blood manganese concentrations and MDI/PDI*. At delivery, the arithmetic mean maternal blood manganese concentration was 22.5 ± 6.5 μg/L and the median was 21.3 μg/L. At 6 months of age, the mean BSID-II scores were 94.4 ± 11.7 and 93.4 ± 14.3 for MDI and PDI, respectively (Table 1). We observed no significant sex differences in BSID-II (data not shown).

**Figure 1:**
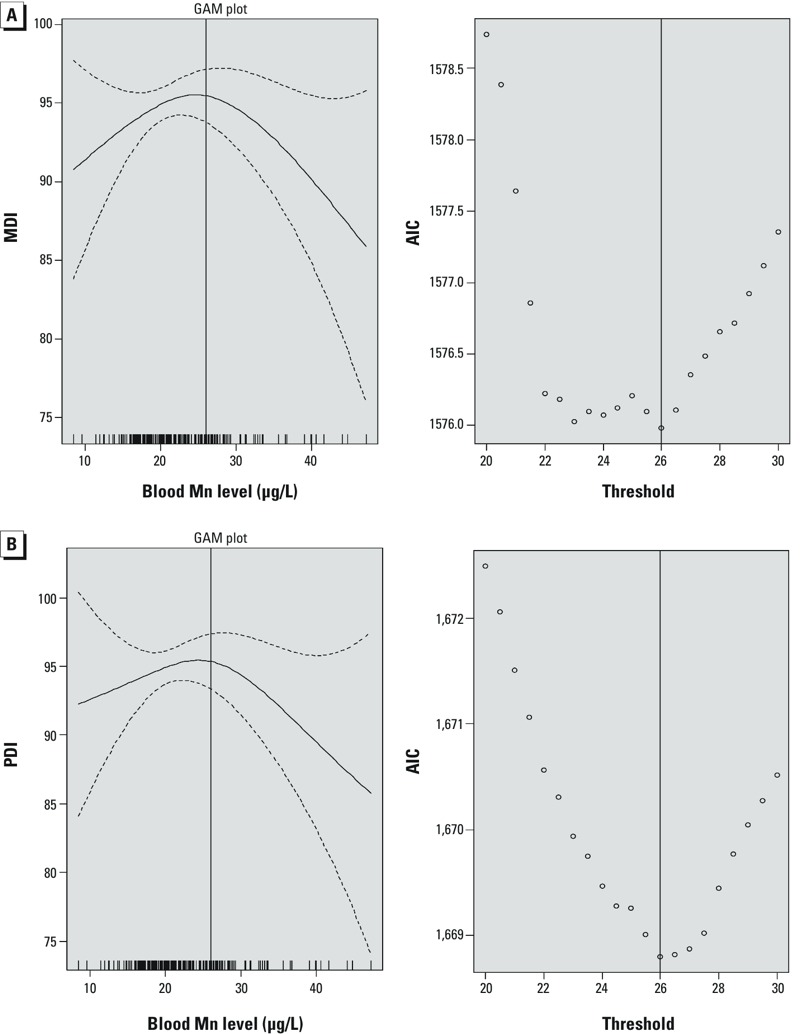
Association between maternal blood manganese (Mn) and (*A*) MDI and (*B*) PDI at 6 months of age and AIC by inflection point. The GAM plots are penalized spline blood manganese (μg/L) predicting 6-month MDI, after controlling for maternal age, gestation period (days), monthly income, breastfeeding status, maternal total calorie intake (kcal/day), infant birth order, residential area, infant sex, and birth weight (kg) among 232 children. The solid line represents the estimate; dotted lines represent the 95% CIs. Vertical lines on the *x*-axis represent the distribution of blood manganese observations. Panels on the right represent the AIC values by moving inflection point per 1 blood manganese concentration (μg/L) with the circles, and the solid line represents the maternal blood manganese concentration with the smallest AIC value.

Using penalized splines of manganese, we observed nonlinear inverted U-shaped associations between blood manganese and 6-month MDI and PDI scores (Figure 1). We also evaluated models of manganese as a simple linear term and confirmed significant improvements in fit after a quadratic term (manganese^2^) was added to the model [MDI: likelihood-ratio test (LRT) *p* = 0.075, PDI: LRT *p* = 0.038]. Based on the smoothed models with the smallest AIC values, associations with increasing concentrations of maternal blood manganese changed from positive to negative at 24–26 μg/L blood manganese for the MDI and at 26–28 μg/L blood manganese for the PDI (Figure 1).

**Figure 2:**
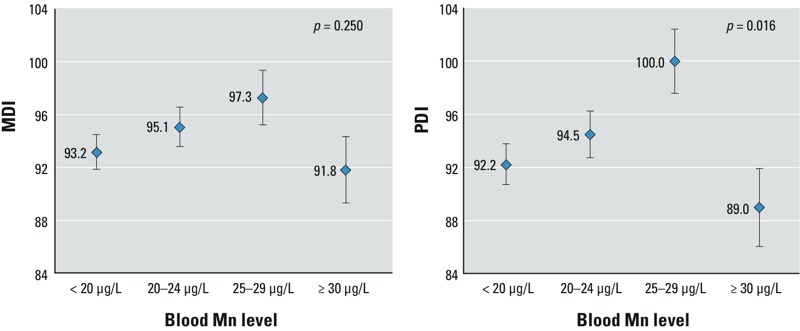
Models of MDI/PDI and maternal blood manganese levels at term, least-square mean after adjusting for maternal age (years), gestation period (days), monthly income, breastfeeding status, maternal total calorie intake (kcal/day), infant birth order, residential area, infant sex, and birth weight (kg). The ranges of blood manganese levels at term were < 20 μg/L (*n *= 82), 20–24 μg/L (*n *= 64), 25–29 μg/L (*n *= 34), and ≥ 30 μg/ L (*n *= 23), respectively. Error bars represent standard errors.

We used separate linear regression models to estimate associations between maternal blood manganese categorized according to four concentration groups (< 20 μg/L, 20.0–24.9 μg/L, 25.0–29.9 μg/L, and ≥ 30.0 μg/L) and MDI or PDI. Models of maternal blood manganese as a categorical variable also indicated an inverted U-shaped dose response (Figure 2). The adjusted mean PDI significantly differed across manganese concentration groups, after adjusting for potential confounders (total model, *p* = 0.016), but MDI did not differ by manganese concentration (total model, *p* = 0.250) (Figure 2). Children from the group with maternal blood manganese of 25.0–29.9 μg/L demonstrated higher 6-month neurodevelopmental scores (MDI and PDI) compared with children in the highest manganese group (≥ 30.0 μg/L) and the lowest manganese group (< 20 μg/L) (mean MDI scores = 93.2 ± 1.3, mean PDI scores = 92.2 ± 1.5). The PDI scores increased 7.8 points until maternal blood manganese reached 25.0–29.9 μg/L (mean PDI scores = 100.0 ± 2.4) and declined sharply by 11.0 points over the highest maternal blood manganese level (≥ 30.0 μg/L) (mean PDI scores = 89.0 ± 2.9). The MDI scores increased 4.1 points until blood manganese level reached 25.0–29.9 μg/L (mean MDI scores = 97.3 ± 2.0) and declined by 5.4 points at the highest blood manganese level (≥ 30.0 μg/L) (mean MDI scores = 91.8 ± 2.5) (Figure 2).

## Discussion

Our findings suggest an inverted U-shaped relation between maternal blood manganese at term and neurodevelopmental indexes of infants 6 months after birth. Increasing maternal blood manganese concentrations up to approximately 24–28 μg/L were positively associated with 6-month PDI scores, whereas higher blood manganese concentrations were associated with lower PDI scores, suggesting adverse neurodevelopmental effects of both low (< 20.0 μg/L) and high (≥ 30.0 μg/L) maternal blood manganese levels. Another study on blood manganese and neurodevelopment found the association to be more prominent for motor function (PDI) than for cognitive function (MDI) for infants 6 months of age (Zoni and Lucchini 2013). Our results are compatible with previous reports in that maternal blood manganese level has biphasic dose–response relationship with the early stage of infant (Claus Henn et al. 2010).

Unlike other toxic heavy metals, manganese is an essential nutrient for human growth and development, having a positive association on human growth and cognitive functions up to a certain level, whereas lead and mercury showed negative associations on cognitive function in children even at low-level exposures (Axelrad et al. 2007). Similar to the results of this study, a cohort study conducted in Mexico City examined the relationships of children’s blood manganese concentrations on their cognitive ability at 12 and 24 months of age, and showed that high blood manganese level during early-life stages was related with impaired neurodevelopment (Claus Henn et al. 2010, 2012). A prospective study conducted in France reported that cord blood manganese concentrations were negatively associated with attention and nonverbal memory scores at 3 years of age, but not significantly associated with neurodevelopmental outcomes at 9 months or 6 years of age (Takser et al. 2003). A recent study reported that *in utero* co-exposure to environmental manganese and lead in the cord blood was negatively associated with neurodevelopment at 2 years of age (Lin et al. 2013).

Biphasic association of maternal blood manganese level was also evident in growth of the fetus. In a cohort of Iranian children, maternal blood manganese was associated with a lower prevalence of intrauterine growth retardation, whereas cord blood manganese was associated with a significantly higher prevalence of intrauterine growth retardation (Vigeh et al. 2008). Zota et al. (2009) reported an inverted U-shape association between maternal blood manganese concentration at term and birth weight, though there was no association between cord blood manganese and birth weight. As the blood manganese concentration reached a certain level, the birth weight started to decrease, demonstrating an inverted U-shape curve as a whole.

Previous research on manganese (Takser et al. 2003; Zota et al. 2009) observed that the average manganese concentration of pregnant women was higher than 20.0 μg/L. Higher blood level during pregnancy may reflect increased physiological demands for fetal and neonatal development (Hatano et al. 1983; Tholin et al. 1995). The Korea National Health and Nutrition Examination Survey 2008 (Korea Centers for Disease Control and Prevention 2008) reported that in the general Korean population, the average blood manganese concentration was higher for adult women (15.1 μg/L) than for adult men (12.3 μg/L). The average blood manganese concentration reported for adult Canadian women was 10.4 μg/L (Health Canada 2010). In contrast with mean blood manganese levels reported for all adult women, mean maternal blood manganese concentrations of 20.4 μg/L (Takser et al. 2003) and 24.0 μg/L (Zota et al. 2009) have been reported at the time of delivery, consistent with the mean maternal blood concentration in our study population (22.5 μg/L). Mean blood manganese concentrations in cord blood appear to be higher, with reported means ranging from 34.3 to 45.0 μg/L (Smargiassi et al. 2002; Takser et al. 2003, 2004; Vigeh et al. 2008; Zota et al. 2009). According to a study in which adults were administered an oral dose of 1.0 mg/day of manganese, women showed higher manganese levels in their blood compared with males (Finley et al. 1994).

Manganese is essential in physical development, infant bone formation, and nutrient metabolism (Aschner and Aschner 2005; Boyes 2010; Yoon et al. 2011). Our study showed that maternal blood manganese level has an association with infants’ neurodevelopment in nonlinear fashion.

A previous study regarding the relationship between children’s blood manganese concentrations and mental development score of 12-month-old babies reported a similar peak point of 24.4 μg/L (Claus Henn et al. 2010). Even though the children’s age and participants were different in our Bayley studies, the concentrations at which associations changed from positive to negative were similar. Neurodevelopment before 24 months of age involves basic activities related to walking and speaking, and it may also be associated with future neurodevelopment, exerting effects even in adolescence and adulthood (Beaudin et al. 2013). Therefore, it is possible that alterations of neurodevelopment associated with exposure to manganese might still be present as the child matures.

Our study has several limitations. First, although the study participants were recruited from the general population, they were not representative of the population as a whole, indicating a potential selection bias. Study participants were recruited typically from metropolitan areas, had higher educational level, and were of mid-range socioeconomic status. Given the relatively high education level of the mothers in our study population, Bayley scores may have been higher than in the general Korean population. However, although this may affect generalizability, it would not be expected to bias the estimated association between maternal blood manganese level and Bayley scores. Second, manganese concentration was measured only at delivery, cord blood and infant manganese concentrations were not measured. Third, environmental exposure and manganese intake from other dietary sources such as soy milk and oral supplements have not been measured. Fourth, although children were followed up after 6 months, there were not sufficient numbers with data for maternal blood manganese concentrations at delivery to estimate associations with neurodevelopmental scores at 12 or 24 months. Therefore, we could not analyze the later relationship between blood manganese and Bayley score. Fifth, there could be additional bias due to restriction of the subjects to term infants and adjustment for gestational age. Gestational age could be an intermediate variable between maternal blood manganese level and neurodevelopment. Although we did not observe differences in adjustment for gestational age among this sample of subjects restricted to term births (data not shown), it is possible that a larger impact of manganese might have been reflected among preterm births; however, the mean manganese levels did not differ among term (22.5 μg/L) versus preterm (22.1 μg/L) infants of excluded participants with < 37 weeks gestation in this study. Finally, we did not investigate potential confounding for other social and environmental factors or genetic influences.

To our knowledge, this is the first prospective epidemiologic study to report an association between maternal blood manganese at delivery and neurodevelopmental scores at 6 months of age in the infants. We detected negative associations between both higher and lower levels of maternal blood manganese and neurodevelopment at 6 months of age. In our study population, mean neurodevelopmental scores were highest among infants whose mothers had blood manganese concentrations between 24 and 28 μg/L at the time of delivery. Few studies have evaluated associations of manganese with psychomotor development in early childhood (Lucchini et al. 2012; Zoni and Lucchini 2013), but high exposure to manganese has been associated with lower motor function in adults with occupational exposure (Lucchini et al. 1999; Shin et al. 2007) and in animal studies (Beaudin et al. 2013). Our findings suggest that *in utero* environment should be taken into account in assessment of postnatal neurodevelopment.

## Conclusion

In this study of term infants in Korea, the association between maternal manganese concentration and neurodevelopment transitioned from positive to negative as maternal blood manganese concentration reached 24–28 μg/L. The relationship was more pronounced for motor function (PDI) than for cognitive function (MDI). Additional research on nonlinear dose–response relationships between metals such as manganese and development of the central nervous system is needed.

## References

[r1] Agency for Toxic Substances and Disease Registry. (2012). Toxicological Profile for Manganese.. http://www.atsdr.cdc.gov/toxprofiles/tp151.pdf.

[r2] American Educational Research Association, American Psychological Association, National Council on Measurement in Education. (1999). The Standards for Educational and Psychological Testing.

[r3] Aschner JL, Aschner M (2005). Nutritional aspects of manganese homeostasis.. Mol Aspects Med.

[r4] Axelrad DA Bellinger DC Ryan LM Woodruff TJ 2007 Dose–response relationship of prenatal mercury exposure and IQ: an integrative analysis of epidemiologic data. Environ Health Perspect 115 609 615; 10.1289/ehp.9303 17450232 PMC1852694

[r5] Bayley N. (1993). Bayley Scales of Infant Development, 2nd ed..

[r6] Beaudin SA, Nisam S, Smith DR (2013). Early life versus lifelong oral manganese exposure differently impairs skilled forelimb performance in adult rats.. Neurotoxicol Teratol.

[r7] Bhang SY, Cho SC, Kim JW, Hong YC, Shin MS, Yoo HJ (2013). Relationship between blood manganese levels and children’s attention, cognition, behavior, and academic performance—a nationwide cross-sectional study.. Environ Res.

[r8] Bouchard M Laforest F Vandelac L Bellinger D Mergler D 2007 Hair manganese and hyperactive behaviors: pilot study of school-age children exposed through tap water. Environ Health Perspect 115 122 127; 10.1289/ehp.9504 17366831 PMC1797845

[r9] Bouchard MF Sauvé S Barbeau B Legrand M Brodeur MÈ Bouffard T 2011 Intellectual impairment in school-age children exposed to manganese from drinking water. Environ Health Perspect 119 138 143; 10.1289/ehp.1002321 20855239 PMC3018493

[r10] Boyes WK (2010). Essentiality, toxicity, and uncertainty in the risk assessment of manganese.. J Toxicol Environ Health A.

[r11] Claus Henn B, Ettinger AS, Schwartz J, Téllez-Rojo MM, Lamadrid-Figueroa H, Hernández-Avila M (2010). Early postnatal blood manganese levels and children’s neurodevelopment.. Epidemiology.

[r12] Claus Henn B Schnaas L Ettinger AS Schwartz J Lamadrid-Figueroa H Hernández-Avila M 2012 Associations of early childhood manganese and lead coexposure with neurodevelopment. Environ Health Perspect 120 126 131; 10.1289/ehp.1003300 21885384 PMC3261931

[r13] Erikson KM, Thompson K, Aschner J, Aschner M (2007). Manganese neurotoxicity: a focus on the neonate.. Pharmacol Ther.

[r14] Finley JW, Johnson PE, Johnson LK (1994). Sex affects manganese absorption and retention by humans from a diet adequate in manganese.. Am J Clin Nutr.

[r15] Guilarte TR 2013 Manganese neurotoxicity: new perspectives from behavioral, neuroimaging, and neuropathological studies in humans and non-human primates. Front Aging Neurosci 5 23; 10.3389/fnagi.2013.00023 23805100 PMC3690350

[r16] Hastie T, Tibshirani R (1990). Exploring the nature of covariate effects in the proportional hazards model.. Biometrics.

[r17] Hatano S, Nishi Y, Usui T (1983). Erythrocyte manganese concentration in healthy Japanese children, adults, and the elderly, and in cord blood.. Am J Clin Nutr.

[r18] Health Canada. (2010). Report on Human Biomonitoring of Environmental Chemicals in Canada: Results of the Canadian Health Measures Survey Cycle 1 (2007–2009). Ottawa, Ontario:Health Canada.. http://www.hc-sc.gc.ca/ewh-semt/pubs/contaminants/chms-ecms/index-eng.php.

[r19] Khan K, Wasserman GA, Liu X, Ahmed E, Parvez F, Slavkovich V (2012). Manganese exposure from drinking water and children’s academic achievement.. Neurotoxicology.

[r20] Kim BM, Ha M, Park HS, Lee BE, Kim YJ, Hong YC (2009). The Mothers and Children’s Environmental Health (MOCEH) study.. Eur J Epidemiol.

[r21] Kim JW, Kim Y, Cheong HK, Ito K (1998). Manganese induced parkinsonism: a case report.. J Korean Med Sci.

[r22] Korea Centers for Disease Control and Prevention. (2008). Korea National Health and Nutrition Examination Survey (KNHANES) Homepage. Ministry of Health and Welfare, Republic of Korea.. http://knhanes.cdc.go.kr.

[r23] KSLM (Korean Society for Laboratory Medicine). (2010). The Korean Society for Laboratory Medicine Homepage.. https://kslm.org/eng/index.php.

[r24] Lin CC, Chen YC, Su FC, Lin CM, Liao HF, Hwang YH (2013). *In utero* exposure to environmental lead and manganese and neurodevelopment at 2 years of age.. Environ Res.

[r25] Lucchini R, Apostoli P, Perrone C, Placidi D, Albini E, Migliorati P (1999). Long-term exposure to “low levels” of manganese oxides and neurofunctional changes in ferroalloy workers.. Neurotoxicology.

[r26] Lucchini RG, Guazzetti S, Zoni S, Donna F, Peter S, Zacco A (2012). Tremor, olfactory and motor changes in Italian adolescents exposed to historical ferro-manganese emission.. Neurotoxicology.

[r27] Menezes-Filho JA, Novaes CO, Moreira JC, Sarcinelli PN, Mergler D (2011). Elevated manganese and cognitive performance in school-aged children and their mothers.. Environ Res.

[r28] Meyer-Baron M, Schäper M, Knapp G, Lucchini R, Zoni S, Bast-Pettersen R (2013). The neurobehavioral impact of manganese: results and challenges obtained by a meta-analysis of individual participant data.. Neurotoxicology.

[r29] Park HW, Cho BH. (2006). Korean Bayley Scales of Infant Development. 2nd ed. Interpretation Manual.

[r30] R Core Team. (2014). R: A Language and Environment for Statistical Computing. Vienna, Austria:R Foundation for Statistical Computing.. http://R-project.org.

[r31] Seber GAF, Wild CJ. (2003). Measures of curvatures and nonlinearity. In: Nonlinear Regression.

[r32] Shin YC, Kim EA, Cheong HK, Cho S, Sakong J, Kim KS (2007). High signal intensity on magnetic resonance imaging as a predictor of neurobehavioral performance of workers exposed to Mn.. Neurotoxicology.

[r33] Smargiassi A, Takser L, Masse A, Sergerie M, Mergler D, St-Amour G (2002). A comparative study of manganese and lead levels in human umbilical cords and maternal blood from two urban centers exposed to different gasoline additives.. Sci Total Environ.

[r34] Spencer A (1999). Whole blood manganese levels in pregnancy and the neonate.. Nutrition.

[r35] Takser L, Mergler D, de Grosbois S, Smargiassi A, Lafond J (2004). Blood manganese content at birth and cord serum prolactin levels.. Neurotoxicol Teratol.

[r36] Takser L, Mergler D, Hellier G, Sahuquillo J, Huel G (2003). Manganese, monoamine metabolite levels at birth, and child psychomotor development.. Neurotoxicology.

[r37] Tholin K, Sandström B, Palm R, Hallmans G (1995). Changes in blood manganese levels during pregnancy in iron supplemented and non supplemented women.. J Trace Elem Med Biol.

[r38] Vigeh M, Yokoyama K, Ramezanzadeh F, Dahaghin M, Fakhriazad E, Seyedaghamiri Z (2008). Blood manganese concentrations and intrauterine growth restriction.. Reprod Toxicol.

[r39] Wasserman GA Liu X Parvez F Ahsan H Levy D Factor-Litvak P 2006 Water manganese exposure and children’s intellectual function in Araihazar, Bangladesh. Environ Health Perspect 114 124 129; 10.1289/ehp.8030 16393669 PMC1332667

[r40] Woolf A, Wright R, Amarasiriwardena C, Bellinger D (2002). A child with chronic manganese exposure from drinking water.. Environ Health Perspect.

[r41] Wright RO, Amarasiriwardena C, Woolf AD, Jim R, Bellinger DC (2006). Neuropsychological correlates of hair arsenic, manganese, and cadmium levels in school-age children residing near a hazardous waste site.. Neurotoxicology.

[r42] Yoon M, Schroeter JD, Nong A, Taylor MD, Dorman DC, Andersen ME (2011). Physiologically based pharmacokinetic modeling of fetal and neonatal manganese exposure in humans: describing manganese homeostasis during development.. Toxicol Sci.

[r43] Zoni S, Lucchini RG (2013). Manganese exposure: cognitive, motor and behavioral effects on children: a review of recent findings.. Curr Opin Pediatr.

[r44] Zota AR, Ettinger AS, Bouchard M, Amarasiriwardena CJ, Schwartz J, Hu H (2009). Maternal blood manganese levels and infant birth weight.. Epidemiology.

